# Highly divergent apicomplexan cytoskeletons provide additional models for actin biology

**DOI:** 10.1111/febs.70263

**Published:** 2025-09-13

**Authors:** Yukino Kobayashi, Ross G. Douglas

**Affiliations:** ^1^ Biochemistry and Molecular Biology, Interdisciplinary Research Centre and Molecular Infection Biology, Biomedical Research Centre Seltersberg, Justus Liebig University Giessen Germany; ^2^ Institute of Veterinary Physiology and Biochemistry Justus Liebig University Giessen Germany

**Keywords:** actin, actin‐binding proteins, apicomplexan, cytoskeleton, *Plasmodium*

## Abstract

Actin is one of the most conserved, abundant and central molecules in eukaryotes. The assembly of monomeric actin into filaments provides the molecular basis for a variety of cellular functions, including muscle contraction, intracellular trafficking, cell shape, division and motility, with classical eukaryotic model systems providing critical insights into these processes. Apicomplexan parasites are a phylum of unicellular eukaryotes with complex life cycles and highly divergent actin cytoskeletons. Their divergent sequences and structures result in overlapping yet different biochemical properties compared to classical systems, providing the opportunity to gain insight into the breadth of actin function and regulation by its binding proteins. These divergent systems also allow the opportunity to identify key sequence determinants for specific biochemical functions. In this review, we highlight the remarkable divergence of the actin cytoskeleton by comparing apicomplexan to classical cell systems, emphasising the valuable knowledge gained by studying these systems to advance our understanding of actin biology across eukaryotes.

## Graphical abstract

Actin is a highly conserved, abundant and central molecule in eukaryotes. Apicomplexans contain some of the most sequence‐divergent actin cytoskeletons known. This divergence results in overlapping yet different biochemical properties. Here, we highlight the remarkable divergence of the actin cytoskeleton by comparing apicomplexan to classical systems, emphasising the valuable knowledge gained by studying these systems to advance our understanding of actin biology across eukaryotes.

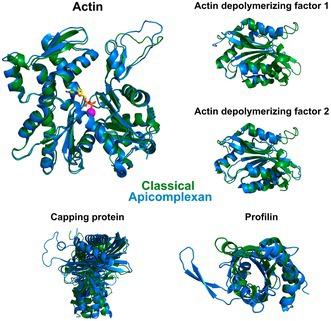

**Abbreviations**
ABP: actin‐binding protein
AC: accession code
ADF: actin‐depolymerising factor
ADP: adenosine diphosphate
ATP: adenosine triphosphate
CAP: cyclase‐associated protein
CARP: C‐terminal X‐linked retinitis pigmentosa two protein
D‐loop: DNase I‐binding loop
EM: electron microscopy
F‐actin: filamentous actin
G‐actin: globular actin

## Introduction

Actin is one of the most highly conserved and abundantly expressed proteins in eukaryotes [1, 2, 3]. It plays a pivotal role in the biology of the cell, serving as a major component of the cytoskeleton. The centrality of actin in cell biology is best highlighted in the variety of roles it plays in fundamental processes such as muscle contraction, cell shape, division, intracellular trafficking, transcriptional regulation and cell motility [4, 5, 6]. The consequences of reduced functional actin due to mutations are seen in actinopathies, which result in muscle diseases, immunocompromised status, intellectual disability, hearing loss, heart and renal defects and neuronal migration defects [7, 8, 9]. In the context of infection, the host actin cytoskeleton is also relied on by pathogens, with *Listeria* bacteria and vaccinia virus both exploiting modulation of host actin to infect and spread between cells [10, 11].

Apicomplexan parasites are a diverse phylum of unicellular eukaryotes with complex life cycles and highly divergent actin cytoskeletons [12, 13]. All apicomplexans are obligatory parasites and include infamous members such as *Plasmodium* (the malaria‐causing parasite), *Toxoplasma* (the causative agent of Toxoplasmosis and one of the most prevalent parasites known), *Cryptosporidium* (the causative agent of the potentially dangerous diarrheal disease Cryptosporidiosis), *Theileria* (the causative agent of tick‐borne Theileriosis in cattle) and *Babesia* (the causative agent of tick‐borne Babesiosis in dogs). These parasites have different shapes and sizes at different life cycle stages, and need to replicate and spread rapidly in very different tissues, often in two very different host organisms that possess different immune responses, body temperatures and metabolic profiles. For example, *Plasmodium* replicates in host liver, blood and the mosquito midgut, needs to be able to invade these tissues and move in the mammalian host skin and the mosquito midgut [14]. Apicomplexans perform cell division differently compared to classical cells [15] and motile stages often move with an uncommon form of actin‐based motility called gliding [16]. These parasites thus need a highly adaptable actin cytoskeleton to cope with all of their needs and dynamic environmental changes. In addition, apicomplexan systems represent some of the most ancient cell biology mechanisms and could exemplify some of the most fundamental eukaryotic mechanisms of the cytoskeleton.

The goal of this review is to highlight the remarkable divergence of the actin cytoskeleton by comparing apicomplexan to classical systems. First, a concise general introduction to actin of classical systems is provided covering their general properties, cellular functions and regulation. Secondly, using a similar framework, what is known about actin in the context of selected apicomplexan parasites is presented and compared to the classical systems. As seen below, the actin cytoskeletons of these parasites possess notable divergence from classical, relatively well‐characterised eukaryotic opisthokont cellular systems (which ranges from yeast to human). Their divergent sequences and structures result in overlapping yet different biochemical properties compared to classical systems, providing the opportunity to gain insight into the breadth of actin function and regulation by its binding proteins, emphasising the valuable knowledge gained by studying these systems to advance our understanding of actin biology across eukaryotes.

## Classical opisthokont actin: General and biochemical properties

**Fig. 1:**
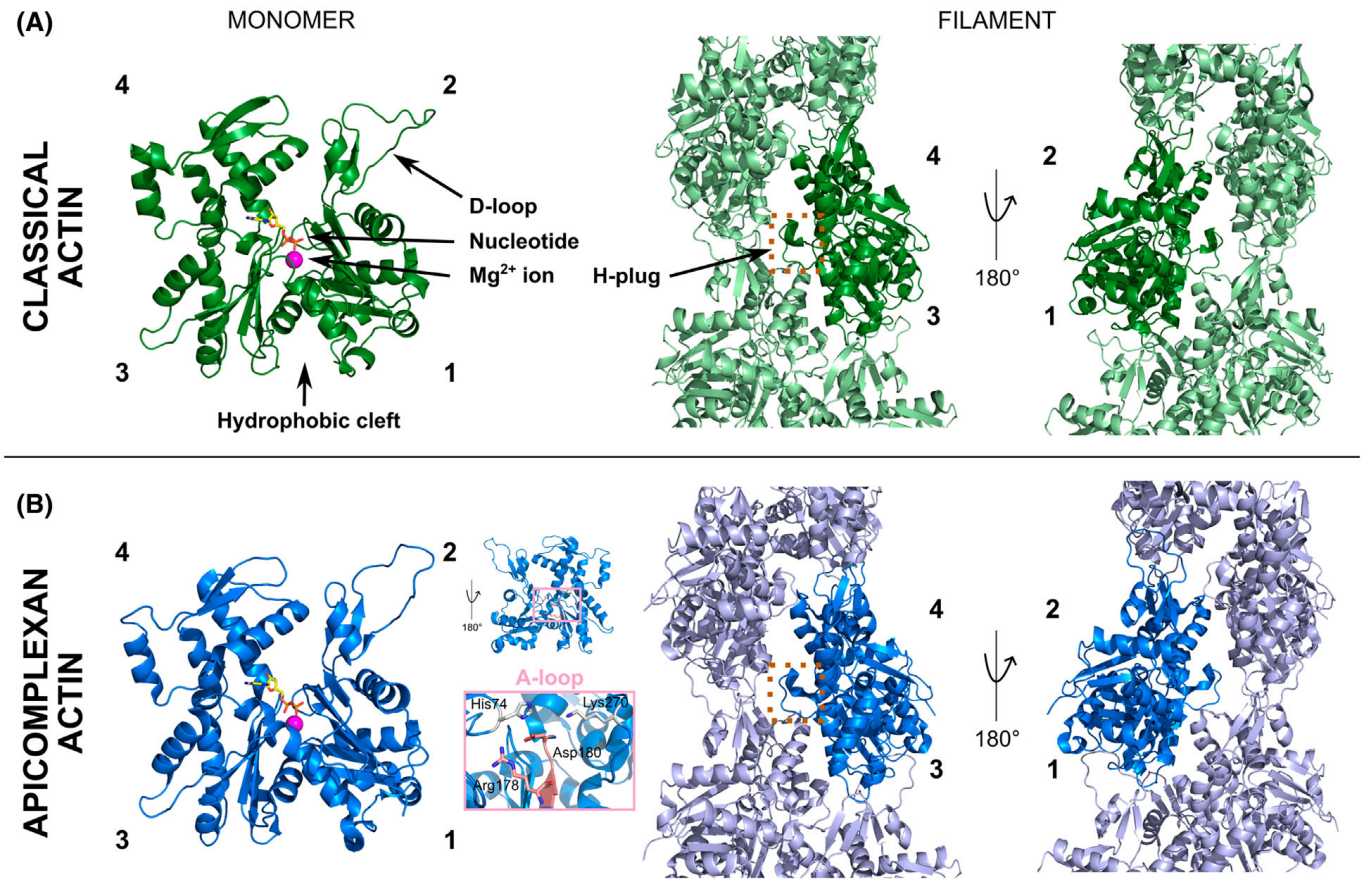
An overview of structural features of the actin monomer and filament for representative classical and apicomplexan actins. (A) Structures of a classical monomer and its position within an actin filament (rabbit skeletal muscle actin, PDB ID: 6t23). The locations of the D‐loop, magnesium ion, nucleotide (ADP) and hydrophobic cleft are indicated on the monomer structure; the H‐plug is indicated in the filament with the dotted gold box. Subdomains 1–4 are indicated in bold text. Visible subdomains are shown for each perspective of the filament. (B) Structures of an apicomplexan monomer and its position within an actin filament (*Plasmodium* actin 1, PDB ID: 6tu4). The relative locations of the D‐loop, magnesium ion, nucleotide (ADP), hydrophobic cleft, H‐plug and subdomains are similar to classical actins. The apicomplexan‐specific A‐loop and associated residues are shown in pink with important interaction residues shown in silver within the pink inset. Images rendered in open‐source PyMOL.

Actin exists in two states termed monomeric globular (G)‐actin and polymeric filamentous (F)‐actin. In many cellular activities, F‐actin takes direct roles as a part of the actin cytoskeleton [6], while G‐actin serves as a building material for F‐actin, strongly depending on the concentration and the bound adenosine nucleotide state [17]. The protein structure of G‐actin is classically divided into four subdomains, whose folding results in a formation of two major clefts, the hydrophobic cleft (between subdomains 1 and 3) and the nucleotide‐binding cleft (between subdomains 2 and 4), representing the classical actin fold [2, 18] (Fig. 1A). The hydrophobic cleft is a common interaction site for many actin‐binding proteins (ABPs) and some polymerisation sequestering toxins [19, 20, 21]. The nucleotide‐binding cleft accommodates ATP or ADP in a divalent cation (Ca^2+^/Mg^2+^) dependent manner, which also influences F‐actin nucleation and affects the activity of selected ABPs [17, 18, 22, 23, 24, 25]. The DNase I‐binding loop (D‐loop) [18], located at the outer corner of subdomain 2, is responsible for the stable longitudinal inter‐subunit linkage in F‐actin [26, 27, 28, 29] (Fig. 1A). While the D‐loop has been shown to adopt multiple structural states during the transition from G‐ to F‐actin [30], the open conformation is most favoured upon polymerisation [27]. Between subdomains 3 and 4, the so‐called H‐plug (originally coined the hydrophobic plug) [26, 31] also contributes to actin filament stabilisation through lateral contacts with the D‐loop and subdomain 3 of the adjacent actin subunit [2, 29, 32] (Fig. 1A).

The polymerisation of actin is a dynamic process involving the sequential association of G‐actin into filaments and consists of two main phases: nucleation and elongation [33]. Actin filament nucleation is activated by the accumulation of G‐actin above a critical concentration [34]. Nucleation follows with the formation of unstable dimers and trimers, followed by more stable oligomers, which then begin to elongate asymmetrically at either end depending on the concentration and nucleotide state of the available G‐actin [35]. In the *in vitro* context with pure actin, the incorporation of ATP‐bound monomers occurs at a faster rate at the growing ‘barbed end’ of the filament. Conversely, ADP‐bound monomers, which are derived from the hydrolysis of ATP once the actin is incorporated into the filament, are predominantly found at the dissociating ‘pointed end’ [17, 33]. The equilibrium between the net addition and loss of monomers at the barbed and pointed ends, respectively, is known as steady‐state ‘treadmilling’ [36, 37]. The structure of actin filament can be described either as a double‐stranded (two‐start) right‐handed long‐pitch helix or a left‐handed helix with a single (one‐start) helix encompassing all monomers within the filament [2, 33, 38]. In the one‐start helix, a monomer within the filament has a twist of between −166.4° and −166.7° relative to the neighbouring monomer [25, 29, 39].

In many classical eukaryotic systems, the dynamic assembly and disassembly result in the formation of long (>1 μm) filaments that are relatively resistant to fragmentation [40, 41] (Table 1). A variety of toxins are able to negatively affect actin polymerisation and depolymerisation. For example, jasplakinolide induces polymerisation and stabilises filaments [42], cytochalasin D primarily binds to barbed ends, preventing elongation and ultimately disrupting filament dynamics [43, 44], while latrunculin toxins bind to actin monomers and prevent polymerisation [45]. Opisthokonts display a range of isoforms, with unicellular organisms such as yeasts typically having one *actin* gene, while birds and mammals express six highly sequence‐conserved yet functionally distinct actin isoforms [46].

**Table 1:** Number of isoforms, summary of general biochemical behaviours, UniProt accession codes and Protein Data Bank IDs of actin and actin‐binding proteins in classical mammalian and apicomplexan systems. Only homologues of actin‐binding proteins that have been identified in apicomplexan parasites and curated on the VEuPathDB [200] are included for comparison. Pf = *Plasmodium falciparum*; Pb = *Plasmodium berghei*; Tg = *Toxoplasma gondii*; Cp = *Cryptosporidium pavum*.

Protein	Isoforms			Biochemical behaviour		UniProt AC		PDB ID	
	Classical (mammals)	Plasmodium	Toxoplasma	Classical (mammals)	Apicomplexa	Classical (mammals)	Apicomplexa	Classical (mammals)	Apicomplexa
Actin	6	2	1	Stable filaments Regulation by wider range of ABPs	Unstable filaments due to increased disassembly rates and shorter lengths (actin 1) (observed *in vitro* with purified actin) No filament branching Less responsive to certain classical actin toxins Regulation by fewer classical ABP homologues	*Homo sapiens* α‐Skeletal muscle actin: P68133 α‐Cardiac muscle actin: P68032 α‐Smooth muscle actin: P62736 γ‐Smooth muscle actin: P63267 γ‐Cytoplasmic actin: P63261 β‐Cytoplasmic actin: P60709 *Oryctolagus cuniculus* α‐Skeletal muscle actin: P68135	Actin‐1: Q8I4X0 (Pf) Actin‐2: Q8ILW9 (Pf) Actin: P53476 (Tg)	*Homo sapiens* α‐Skeletal muscle actin: 9DUU β‐cytoplasmic actin: 8DNH *Oryctolagus cuniculus* α‐Skeletal muscle actin: 6T23	Actin‐1: 6TU4 (Pf) Actin‐2: 8CCN (Pf) Actin: 8TRM (Tg)
Profilin	4	1	1	Removes ADP from dissociated monomeric actin Delivers actin monomers to the site of polymerisation Interacts with formin and Ena/VASP	Sequesters monomeric actin	*Homo sapiens* Profilin‐1: P07737 Profilin‐2: P35080 Profilin‐3: P60673 Profilin‐4: Q8NHR9	Profilin: P86294 (Pf) Profilin: Q58NA1 (Tg)	*Homo sapiens* Profilin‐1: 1FIL Profilin‐2: 1D1J	Profilin: 2JKF (Pf) Profilin: 3NEC (Tg)
Coronin	7	1	1	Promotes rapid actin network expansion Interacts with Arp2/3 complex Bundling of filamentous actin	Bundling of actin filaments	*Homo sapiens* Coronin‐1A: P31146 Coronin‐1B: Q9BR76 Coronin‐1C: Q9ULV4 Coronin‐2A: Q92828 Coronin‐2B: Q9UQ03 Coronin‐6: Q6QEF8 Coronin‐7: P57737	Coronin: A0A509ARM7 (Pb) Coronin: Q5Y1E7 (Tg)	*Homo sapiens* Coronin‐1C: 7STY Coronin‐6: 7KYX	Coronin: 4OZU (Tg)
ADF/Cofilin	3	2	1	Promotes dissociation of Pi in actin filament Severing of actin filaments Blocking of nucleotide exchange in monomeric actin	No binding to actin filaments (*P. berghei* homologue 1) Severing of actin filaments (*P. berghei* homologue 1)	*Homo sapiens* Cofilin‐1:P23528 Cofilin‐2:Q9Y281 Destrin: P60981	Cofilin/actin‐depolymerising factor homologue 1: Q8I467 (Pf) Actin‐depolymerising factor 2: Q3YPH0 (Pb) Actin‐depolymerising factor: O15902 (Tg)	*Homo sapiens* Cofilin‐1: 4BEX Cofilin‐2: 7M0G Destrin: 1AK6	Cofilin/actin‐depolymerising factor homologue 1: 2XF1 (Pf) Actin‐depolymerising factor 2: 2XFA (Pb) Actin‐depolymerising factor: 2MOT (Tg)
Formin	≥15	3	3	Recruitment of profilin‐bound monomeric actin to the site of polymerisation Prevents actin filament capping at barbed ends Reorganisation of filamentous actin into linear bundles Interacts with fascin	Nucleates actin filaments in stage‐specific manner Does not cooperate with profilin	*Homo sapiens* Protein diaphanous homologue 1: O60610 Protein diaphanous homologue 2: O60879 Protein diaphanous homologue 3: Q9NSV4 Dishevelled‐associated activator of morphogenesis 1: Q9Y4D1 Dishevelled‐associated activator of morphogenesis 2: Q86T65 Formin‐1: Q68DA7 Formin‐2: Q9NZ56 FH1/FH2 domain‐containing protein 1: Q9Y613 FH1/FH2 domain‐containing protein 3: Q2V2M9 Inverted formin‐2: Q27J81 Formin‐like protein 1: O95466 Formin‐like protein 2: Q96PY5 Formin‐like protein 3: Q8IVF7 Delphilin: A4D2P6 FH2 domain‐containing protein 1: Q9C0D6	Formin 1: A0A509AMX5 (Pb) Formin 2: A0A509AUT2 (Pb) Nuclear formin‐like protein MISFIT: A0A509ANE6 (Pb) Formin 1: D0V3Y0 (Tg) Formin 2: D0V3Y1 (Tg) Formin 3: B6KTA3 (Tg)	*Homo sapiens* Protein diaphanous homologue 1: 8RU2 Protein diaphanous homologue 3: 5UWP Dishevelled‐associated activator of morphogenesis 1: 2Z6E Formin‐2: 3R7G FH1/FH2 domain‐containing protein 1: 3DAD Inverted formin‐2: 9AZ4 Formin‐like protein 1: 4YDH Formin‐like protein 2: 4YC7	ND
Cyclase‐associated proteins	2	1	2	Promotes monomeric actin nucleotide exchange	Promotes monomeric actin nucleotide exchange (although much smaller)	*Homo sapiens* Adenylyl cyclase‐associated protein 1: Q01518 Adenylyl cyclase‐associated protein 2: P40123	Cyclase‐associated protein: Q8I288 (Pf) Cyclase‐associated protein: A0A125YN74 (Tg) Cyclase‐associated protein: Q5CS32 (Cp)	*Homo sapiens* Adenylyl cyclase‐associated protein 1: 1K8F	Cyclase‐associated protein: 2B0R (Cp)
Capping proteins	>3 (alpha subunit)	1 alpha 1 beta	1 alpha 1 beta	Caps the ends of actin filaments Slowing down actin filament depolymerisation	Caps actin filaments Independent expression and functions of alpha and beta subunits	*Homo sapiens* F‐actin‐capping protein subunit Alpha‐1: P52907 F‐actin‐capping protein subunit Alpha‐2: P47755 F‐actin‐capping protein subunit Alpha‐3: Q96KX2 F‐actin‐capping protein subunit beta: P47756	F‐actin‐capping protein subunit alpha: A0A509AR49 (Pb) F‐actin‐capping protein subunit beta: A0A509AQN8 (Pb) Capping alpha‐like subunit: A0A125YI27 (Tg) F‐actin‐capping protein subunit beta: S8EN02 (Tg)	*Homo sapiens* F‐actin‐capping protein subunit Alpha‐1: 8F8Q F‐actin‐capping protein subunit beta: 8F8Q	F‐actin‐capping protein subunit alpha: 7A0H (Pb)
	1 (beta subunit)								

## Selected actin‐dependent cellular functions

Actin is vital for many important cellular functions such as cell motility [6, 47, 48], tissue integrity [49], intracellular transport [4] and cell division [50]. The actin cytoskeleton is a major part of the force‐generating machinery of eukaryotic cells [6] and produces the protrusive structures required for migrating cells either through branched networks (to produce lamellipodia) or linear bundles of actin filaments (to produce filopodia) [51]. These protrusions of the plasma membrane also allow cells to sense and interact with the extracellular environment, as well as capture pathogens for phagocytosis [6, 52]. Local detachment of the plasma membrane from the actomyosin cortex results in spherical membrane expansion [51, 53]. This ‘bleb’ formation is an important mechanism for three‐dimensional cell migration, cytokinesis, tissue morphogenesis and apoptosis [54, 55, 56].

Interactions between actin and myosin superfamily motor proteins are fundamental to many biological functions [48, 57]. During muscle contraction, the motor domain of myosin II interacts with tropomyosin‐decorated actin filaments to conduct power strokes that generate muscle force [58]. Nonmuscle myosin II isoforms also interact with actin filaments and are key drivers of the actomyosin cortical meshwork [59], as well as the bundles of actin stress fibres that enable cells to achieve directional migration by regulating protrusion and adhesion dynamics [60, 61]. In addition to cell shape changes, rapid local assembly and disassembly of actomyosin filaments is likely to support tissue‐wide deformation and actomyosin turnover in epithelial cell morphogenesis [49]. Short‐range intracellular transport of various cellular components such as organelles, vesicles and ribonucleoproteins is mainly mediated by actin and the myosin V motor family [4]. Endocytosis is a complex stepwise process for the internalisation of external and surface biomolecules and is critical for nutrient acquisition, signalling and membrane homeostasis [62]. In clathrin‐mediated endocytosis, myosin I localises to the site of actin polymerisation and is essential for facilitating vesicle budding [63, 64].

Contraction of actomyosin around the nucleus initiates chromatin reorganisation after cell division and can affect gene expression by compressing chromatin as nuclear volume decreases [65, 66]. While such mechanical stiffening can lead to nuclear envelope rupture and subsequent loss of DNA repair factors [67], the assembly of nuclear actin filaments at the site of double‐stranded DNA breaks has also been observed to promote efficient cleavage clearance [68]. Furthermore, the amount of nuclear actin correlates with the transcriptional activity of the cell [69]. Some chromatin remodellers, such as INO80 and SWR1, contain monomeric actin in their complexes together with actin‐related proteins (Arps) [70, 71, 72].

## Conventional regulation of actin dynamics

The involvement of actin in many vital cellular activities underlines the importance of tight regulation of this essential protein. While actin concentration can influence its own polymerisation dynamics [34], the overall architecture and behaviour of the actin cytoskeleton is largely dependent on the binding of ABPs. More than one hundred ABPs are known to regulate actin dynamics and function in opisthokont eukaryotes. ABPs bind both monomeric and/or polymeric actin and regulate all aspects of actin assembly, including nucleation, elongation, filament organisation (such as bundling and crosslinking) and depolymerisation [33, 73]. A selection is discussed here.

Actin monomer binders such as thymosin‐beta and profilin influence the assembly dynamics of monomeric actin. Thymosin‐beta sterically sequesters monomers and prevents their incorporation into filaments at both barbed and pointed ends [33]. Profilin, on the other hand, promotes monomer incorporation at the barbed end by inducing a conformational change in the actin monomer nucleotide‐binding site and thus facilitates a more rapid exchange of ADP to ATP [74, 75]. Profilin also delivers actin monomers to the site of filament elongation by interacting with elongation factors such as formin and Ena/VASP [76, 77, 78, 79]. Cyclase‐associated protein (CAP) also catalyses nucleotide exchange with an even higher binding affinity to ADP‐G‐actin than profilin [23, 78, 79, 80] (Table 1).

Formins and Ena/VASP family proteins promote elongation of linear filaments by recruiting profilin‐bound actin monomers and by protecting barbed ends from being blocked by capping proteins [81, 82, 83]. At filopodial tips, these proteins anchor elongating actin filaments to the membrane and reorganise them into aligned linear bundles to generate protrusive force [6]. Fascin is another essential bundling protein that recruits formin and provides rigidity to the bundles [84, 85]. The branched organisation of cortical F‐actin is particularly important for lamellipodia motility and phagocytosis [77, 86]. Branched actin filaments are primarily initiated by the Arp2/3 complex and its nucleation‐promoting factors such as the WASP family proteins [87]. Coronin bundles filamentous actin as well as promotes rapid actin network expansion by recruiting Arp2/3 complex to nucleation sites, while also acting to mediate filament severing in coordination with the actin‐depolymerising factor ADF/Cofilin at the ADP‐rich pointed end [88] (Table 1).

Suppression of actin filament nucleation and elongation by capping and severing proteins is another important mechanism for regulating actin‐dependent biological processes. Capping proteins can bind to the ends of filaments, preventing their elongation but also potentially slowing their disassembly. Members of the ADF/Cofilin family disassemble the actin network by promoting P_i_‐dissociation from the bound ADP and severing filaments [89] (Table 1). Nucleotide exchange on monomeric actin is also inhibited by ADF/cofilin, which can be competitively compensated for by profilin and CAP [23, 90].

## Apicomplexan actin: General and biochemical properties

**Fig. 2:**
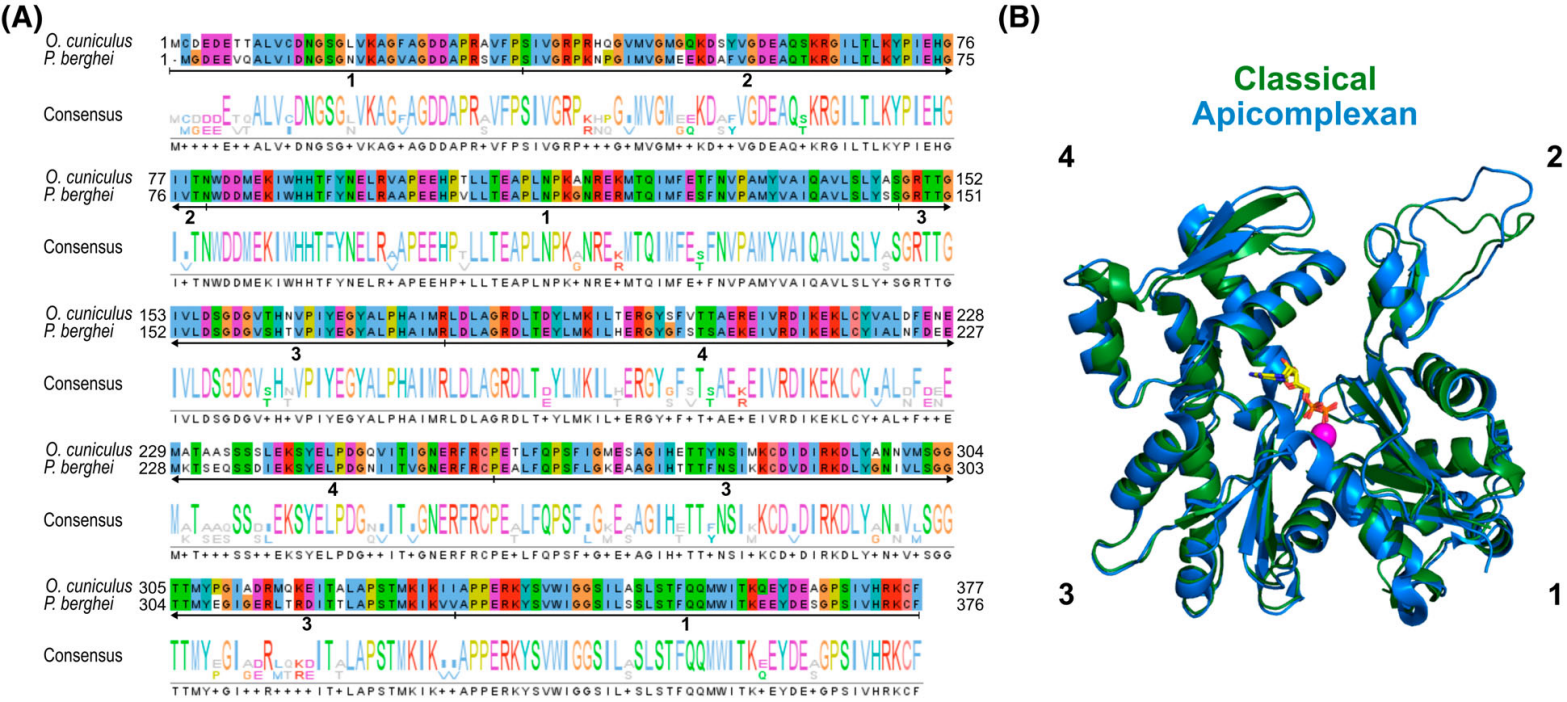
Apicomplexan actin is highly sequence divergent and has a conserved monomeric structure. (A) Sequence alignment of a classical actin (*O. cuniculus* (rabbit) α‐skeletal muscle actin, UniProt AC: P68135) and a representative apicomplexan actin (*P. berghei* actin 1, UniProt AC: Q4Z1L3). The parts of the primary structure forming each of the four subdomains are indicated below the sequences (with a line and corresponding subdomain number). *P. berghei* actin has <82% sequence identity in comparison to conventional actins. Alignment performed using Clustal Omega and visualised with Jalview. (B) Structural alignment of classical actin (rabbit α‐skeletal muscle actin, green, PDB ID: 6t23) and apicomplexan actin (*P. berghei* actin 1, blue, PDB ID: 6tu4) monomers. Subdomains indicated in bold text. The nucleotide (ADP) and magnesium ion are shown as sticks and magenta spheres, respectively. Image rendered in open‐source PyMOL.

Apicomplexan actins represent some of the most divergent eukaryotic actins known when compared to classical systems [38]. When comparing vertebrate actins, there is a very high conservation between different species (100% sequence identity between chicken and human alpha skeletal muscle actins) and isoforms (>93% sequence identity and >96% sequence similarity between rabbit alpha skeletal muscle and beta actins). In contrast, apicomplexan actins typically display <82% sequence identity and 92% sequence similarity when compared to vertebrate actins (Fig. 2A) and also between protists from different clades [91]. This indicates a general divergence of actin that is not apicomplexan specific. These sequence changes have fundamental consequences for actin filament dynamics. Purified *Plasmodium* actin 1 (UniProt AC: Q8I4X0) and *Toxoplasma* actin (UniProt AC: P53476) filaments are inherently unstable *in vitro* (Table 1). This is seen in long timeframe assays, where prolonged incubation ultimately results in only short filaments of approximately 100 nm length [92, 93, 94]. *Plasmodium* actin 1 displays a classical nucleation–elongation mechanism with similar assembly rates compared to classical actins, while *Toxoplasma* actin has a much higher critical concentration and slower assembly rate [95, 96, 97]. In both systems, apicomplexan actins can form long filaments [96, 97], yet disassembly and fragmentation rates are markedly higher in these species [95, 96, 97]. Apicomplexans also display reduced numbers of actin isoforms in comparison to mammals (Table 1), and thus more resemble the number of isoforms found in yeast. Interestingly, the second *Plasmodium* actin isoform (actin 2, UniProt AC: Q4YU79) forms more stable filaments that are more comparable to classical actins indicating that *Plasmodium* needs a more stable filament system at least for certain parts of its life cycle [94].

The divergent sequences of apicomplexan actin are important for their biochemistry and biology. Structurally, apicomplexan actin monomers tertiary structures and filament architectures are largely similar to canonical actins [92, 94, 96] (Figs 1B and 2B), although the *Plasmodium* actin 1 filament strikingly has a larger helical twist by approximately 1° [98]. Yet small changes in lateral and longitudinal contacts, particularly mediated by changes in sequence in subdomains 2, 3 and 4, can have big consequences on filament stability [98]. Mutations in these regions generally lead to the formation of more stable filaments [94, 99, 100]. This divergence is fundamental for the biology of these parasites (see below). Internal dynamics of monomers within the filament are also important. As an example, a divergent subdomain 3 loop (given the name ‘A‐loop’ to indicate its anionic nature, the presence of asparagine and arginine residues in the loop and also the prominence of the loop in apicomplexan actins) has been shown to act as a dynamic sensor for phosphate release and as a switch between stable and unstable filament conformations [100] (Fig. 1B). Bringing this together, structural and mutagenesis studies using apicomplexan actins have begun to reveal the sequence requirements for an actin to form a more or less stable filament. This highlights how highly divergent actins can be used to understand the requirements for stable actin monomer incorporation into filaments, a fundamental question in biology.

The divergence of apicomplexan actins is also highlighted in their responsiveness to small molecules. Typical broad‐spectrum polymerisation inhibitor latrunculin does not impact apicomplexan motility, and the apicomplexan actin cytoskeleton cannot be effectively stained by phalloidin and SiR‐actin [101, 102, 103, 104]. These insensitivities highlight just how divergent these actins are as these actin‐binding compounds work on many different cell types and species. Further, it provides a proof‐of‐concept that one could develop compounds that are selective against actin of a particular species.

While the focus of this review is on apicomplexan actins, it is worth emphasising that the divergent properties of actin are not limited to apicomplexans and are also seen in other unicellular parasites. *Leishmania* actin is also prone to higher depolymerisation rates at both ends of the filament [91] and remarkably has DNA‐nicking activity with kinetoplastid DNA *in vitro* [105], an observation that seems not to be found in other organisms including trypanosomes [106]. *Giardia* actin also produces shorter filaments and even displays highly altered ultrastructural features, with *Giardia* protofilaments being observed [107]. Taken together, protist actin systems across eukaryotes tend to be inherently prone to disassembly and require rapid turnover for their biology.

## Actin‐dependent cellular functions in selected apicomplexan parasites

Gene knockout, gene expression knockdown, gene mutation and actin inhibitor studies have uniformly indicated the essentiality of the major actin isoforms in parasite viability, unsurprisingly given the central nature of actin isoforms already highlighted in classical systems. Apicomplexan's actins, like those of its classical homologues, are involved in multiple processes including specialist functions required for their unique biology. Nonetheless, despite all the differences in actin biochemistries, there are commonalities between these disparate members indicating fundamentally conserved features of actins across eukaryotes, thus highlighting core functions and the sequence determinants for specific biochemical properties.

### Gene expression, nuclear division and cell division

The role of actin is well known to be crucial for the cytokinetic contractile ring in many eukaryotes. Inducible knockout of actin 1 or formin‐2 in early blood stage *Plasmodium* parasites also indicated an important role for actin 1 in this process, although near complete depletion of the protein did not result in a complete block of parasite segmentation [108, 109]. While it has not yet been possible to test the involvement of actin in other replicative stages in *Plasmodium*, it is reasonable to suggest a similar importance in these stages. Interestingly, actin‐dependent lobular structures have been observed in *Theileria annulata* replicative stages (schizonts) [110]. While the functional significance of these structures is still not known, it has been suggested that actin filament protrusions could facilitate optimal positioning for cell and nuclear division [110].

A more recent appreciation of the role of actin in classical cells has been its role in and around the nucleus, where it plays important roles in chromatin remodelling, gene expression, DNA repair as well as nuclear structure and envelope stiffness [65, 111, 112]. Not much is known about the nuclear role of actin in apicomplexan parasites. Chromatin immunoprecipitation studies have indicated a potential role of actin in regulating monoallelic expression of immune evasion genes hinting at a role in chromatin remodelling and gene positioning [113, 114, 115]. There could be interesting alternative roles for parasite cytoskeletal components in apicomplexan parasites. As an illustration, knockout of *P. berghei* alpha‐tubulin 1 did not affect genome replication and nuclear multiplication in mosquito stage oocysts and that these processes occurred in the absence of microtubules at this stage [116]. The replication and multiplication in the absence of microtubules strongly implicates actin as a replacement, a suggestion that is supported by observations for nuclear fission in fission yeast and thus likely an ancient mechanism [117].

### Organelle distribution, trafficking and endocytosis

Distribution of organelles to daughter cells is a fundamental requirement for eukaryotic cell division. In apicomplexans, actin dynamics are important for the distribution of the apicoplast [118, 119, 120], an apicomplexan‐specific plastid that is essential for at least four anabolic pathways [121], but not the mitochondrion [108, 109], suggesting that a different mechanism is employed by apicomplexan parasites in comparison to the classical mechanisms known for mitochondrial fission in mammalian cells [108, 122, 123]. For many years, given the instability of filaments and the difficulties of visualising actin filaments *in vivo*, the role of apicomplexan actins in trafficking and material distribution remained unclear. Visualisation of the actin network in *Toxoplasma* using an actin‐targeting chromobody revealed an elaborate actin‐containing tubule network that connected parasite daughter cells and was critical for organelle distribution [109, 124, 125]. The exact mechanistic basis of how material is distributed and coordinated via this network remains to be determined.

A branched actin network is involved in classical endocytosis. In the case of apicomplexan parasites, the absence of classical branching factors and observable branched filaments indicates that the roles mediated in endocytic pathways are likely done with only unbranched filaments. Some of the first insights of *Plasmodium* actin's *in vivo* role came from inhibitor studies on parasitised red blood cells and revealed a role in endocytic trafficking of haemoglobin [126, 127, 128]. While the exact mechanisms of haemoglobin uptake are still being delineated [129, 130], actin is clearly important for multiple steps of haemoglobin uptake, including the structure of the cytosome [126, 128] and for myosin‐dependent trafficking [131]. Interestingly, filament stabilisation by jasplakinolide generally resulted in more striking effects on particular uptake steps compared to cytochalasin D (which disrupts filament formation) [126, 127, 128], perhaps hinting at an adaptation of trafficking with more dynamic filaments in these parasite‐specialised intracellular processes. While *Toxoplasma* has been unequivocally shown to engage in endocytosis [132, 133], little is currently known about the role of actin in this process. Extracellular tachyzoites also perform endocytosis and, interestingly, a fountain‐flow model has been proposed to allow for efficient adhesion turnover and thus rapid motility parasite, linking an internalisation process with extracellular movement [134].

### Parasite motility and invasion

**Fig. 3:**
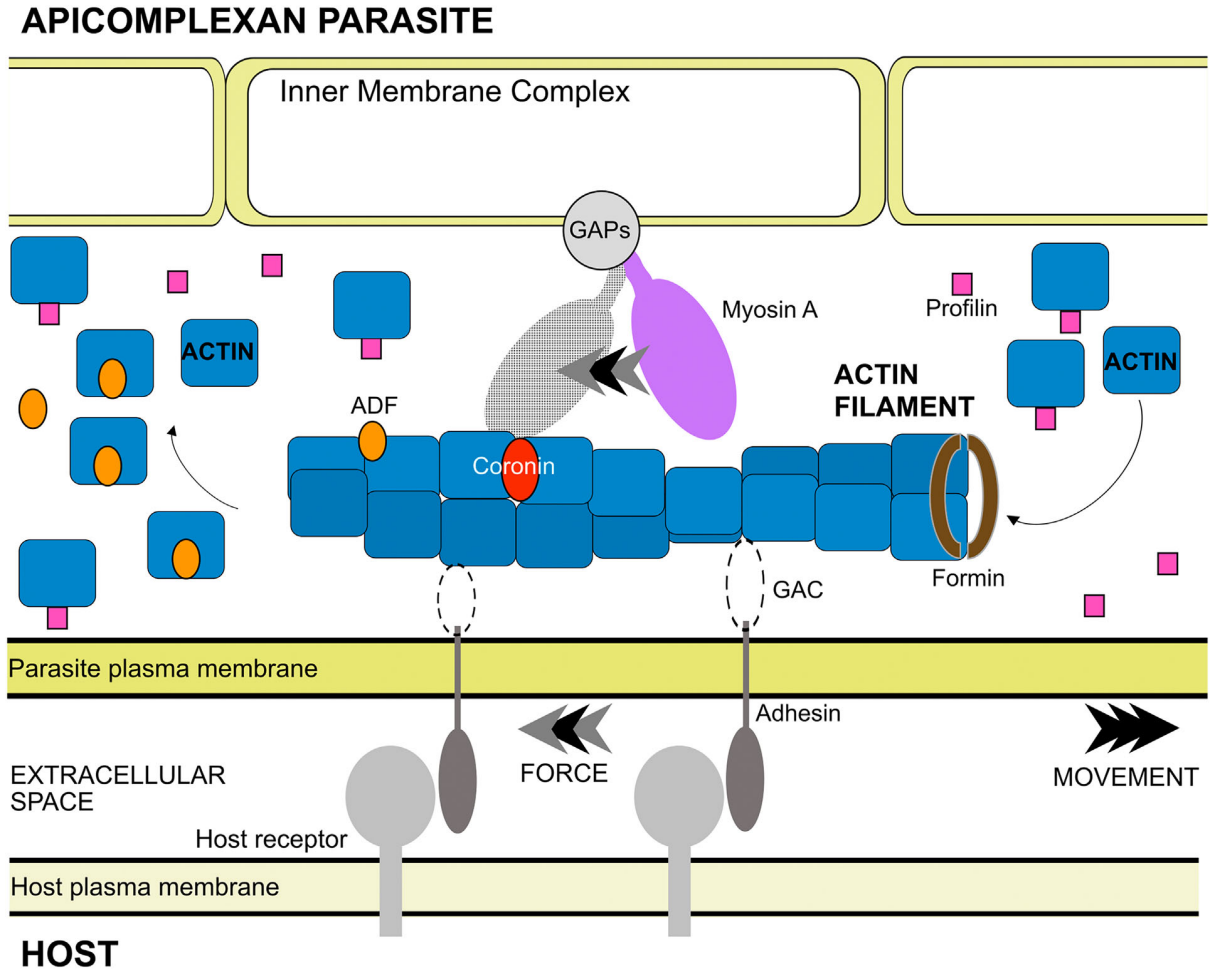
A simplified overview of the actomyosin‐based gliding machinery. The myosin A motor is located on a stable intracellular membranous system directly subtending the plasma membrane (the inner membrane complex). The power stroke of myosin results in the rearward translocation of the actin filament and associated membrane‐spanning adhesin, which ultimately propels the organism forward. Selected actin‐binding proteins are shown. The dynamic turnover of the actin filament forms the central piece of the machinery. For completeness, the location of connectors for the myosin to the inner membrane complex (glideosome‐associated proteins, GAPs) and between the actin filament and adhesin (glideosome‐associated connector, GAC) are also shown.

*Plasmodium* and *Toxoplasma* are known to rely on their active motility powered by the actomyosin motor [14, 135, 136]. The machinery consisting of myosin A motor complex and a dynamic actin filament [137], collectively called the glideosome [138], generates locomotive force that enables parasites to efficiently invade and traverse within their hosts [139, 140]. Apicomplexan parasites rely on an uncommon form of active movement known as gliding motility at particular stages of their life cycles. Gliding speeds of these parasites are remarkably fast—on average about 1–3 μm/s [141, 142], more than an order of magnitude faster than the immune cells that chase them and the winners of the mammalian cell race [143]. A highly coordinated actomyosin system with highly dynamic actin filaments is critical for efficient parasite gliding. In the linear motor model, the force of the power stroke of myosin (an apicomplexan unique class XIV myosin) is transferred through associated actin filaments and membrane‐spanning adhesins. This rearward‐directed locomotive force results in forward propulsion of the organism that enables parasites to efficiently invade different cells and traverse different tissues in their hosts (Fig. 3).

The central importance of actin in parasite gliding was first observed when treating moving parasites with actin inhibitors. Treatment with most classical actin toxins (such as asplakinolide and Cytochalasin D) resulted in an abrupt block of invasion and motility of *Plasmodium*, *Toxoplasma* and *Cryptosporidium* [144, 145, 146, 147, 148, 149]. Interestingly, high concentrations of Latrunculin, an actin monomer binder and polymerisation inhibitor, have no observable effects on both *Toxoplasma* tachyzoites and *Plasmodium* sporozoites gliding indicating that the highly divergent nature of apicomplexan actin renders it insensitive to some compounds. More recently, genetic studies have further highlighted the critical role of actin in gliding motility and invasion. Both overexpression and inducible knockout of *Toxoplasma* actin have shown the important role of actin in gliding motility [119, 120, 150]. Inducible knockout of actin 1 in *P. falciparum* blood stages surprisingly produced a limited amount of daughter cells that could egress the host red blood cell, but none of these actin‐depleted parasites were able to invade new cells [108] highlighting the essential role of actin in the invasion process.

The highly divergent *Plasmodium* actin 1 sequence has been recently explored in detail across the life cycle using the rodent‐infecting malaria parasite *P. berghei*. Certain mutations in different actin subdomains resulted in different effects on mosquito‐stage parasite gliding. Remarkably, the parasite could survive a combined 16 amino acid residue change in subdomain 4, but highly motile and infectious parasites could not move smoothly [137]. Mutations of D‐loop residue Pro42 (mammalian equivalent Gln41) and H‐plug residue Lys270 (mammalian equivalent Met269) in subdomains 2 and 3, respectively, were not tolerated by the parasite [137]. Strikingly, all viable mutations of residues in subdomains 2, 3 and 4 resulted in a drastic reduction in the number of parasites able to invade the mosquito salivary glands specifically, indicating an optimal actin dynamic necessary for penetration of this organ [137, 151]. Further investigations on other apicomplexans will reveal the similarities and differences of actin in gliding and invasion within the phylum. Indeed, the recent development of reverse genetic tools in *Cryptosporidium* will allow for such an analysis of this clinically important parasite [152, 153, 154, 155].

As mentioned above, visualising actin filaments in apicomplexan parasites has historically been elusive and thus there was limited information as to how filaments are located in actively gliding or invading parasites. Traditionally (before 2017), only certain apicomplexan parasites treated with jasplakinolide, under fixed EM conditions, enabled visualisation of actin filaments but not under living conditions [110, 145]. However, in the last few years, technological developments have allowed such a possibility and provided significant insights into this important and rapid process. Filamentous actin in motile and invading parasites was first characterised with the actin chromobody. This revealed distinct locations of actin filaments in the anterior and posterior poles of the moving and invading parasites [151, 156]. Even more recently, native filaments much longer than originally anticipated have been visualised in motile apicomplexan parasites [157, 158]. This allowed for a more exact localisation of filaments, their respective lengths as filaments were propelled back and the role of the rear of the parasites in filament recycling to maintain fast motility. A significant amount of actin filaments in the nucleus was also observed in *Plasmodium* [151, 158]. The functional role of these filaments in a rapidly motile cell is yet to be understood.

There is an interesting difference in the number of actin isoforms between apicomplexans. *Toxoplasma*, for example, codes for one conventional actin in its genome, as do many other apicomplexans. *Plasmodium* on the other hand codes for two conventional actin isoforms (Table 1). Actin 1 is very similar in terms of sequence to *Toxoplasma* actin, is essential for the entire life cycle and cannot be knocked out or accommodate some mutations. Actin 2, which tends to form more stable filaments, on the other hand, can be straightforwardly deleted without any major effects on blood stage growth [159]. However, there is a block in the maturation of male gametes that prevents transmission to the mosquito host which cannot be effectively compensated for by actin 1 [160]. Given the presence of this second isoform, it appears that a second specialist actin is needed for transmission to an arthropod vector. Actin 2 is expressed in later stages as well, but the functional significance of actin 2 at those stages awaits further investigations.

It is worth mentioning that myosin dynamics, crucial for many of the actin‐based processes discussed in this review, are also divergent from conventional myosins. Apicomplexan myosin A is the most conserved, best studied, a central myosin in parasite gliding motility and serves as an example. Myosin A is a class XIV myosin (a class consisting of apicomplexan and ciliate myosins), has a modified neck region and lacks any prominent form of tail [161]. It performs its power stroke with a largely immobile SH1‐helix and thus without the classical piston‐like movement usually observed for classical myosins [162]. Myosin A has a unique N‐terminal extension that is dependent on phosphorylation, which influences ADP release and tunes the motor's properties [162, 163, 164, 165]. This is both interesting and informative, as it indicates the spectrum of stroke that a myosin can make and the sequence contributions required for this.

## Regulation of apicomplexan actin dynamics

Apicomplexans could represent minimalist cytoskeletal regulatory systems since they possess markedly fewer predicted ABP homologues [166]. Indeed, while about 100 opisthokont ABPs have been discovered, apicomplexans only express an order of magnitude less. Notable absentees include thymosin‐beta, fascin, talin and Ena/VASP. Apicomplexans generally also have fewer isoforms (Table 1). Further, classical ABPs found in apicomplexans, while maintaining core functionalities, have altered biochemistries in these parasites (Table 1), presenting an opportunity to determine how sequence alterations contribute to altered biochemical functions or the minimalist requirements for ABP function. As a summary, apicomplexan profilin lacks classical interactions with formins, only sequesters monomers and inhibits nucleotide exchange. Apicomplexan profilin possesses a unique additional arm domain to facilitate specific interactions with parasite monomeric actin, while apicomplexan formins lack classical regulatory features. CAPs are noticeably smaller yet can still mediate nucleotide exchange, while apicomplexan coronin displays only weak filament bundling activity despite a structurally conserved WD40/beta propeller domain. One apicomplexan ADF is one of the smallest known ADFs and lacks a classical F‐actin‐binding loop, which limits the ability to bind F‐actin. Surprisingly, studied *Plasmodium* ADFs stimulate nucleotide exchange rather than inhibit it. The alpha subunit of *Plasmodium* capping protein can remarkably function as an asymmetric homodimer to mediate filament capping. These apicomplexan ABPs are discussed in more detail below.

For nucleation, formin isotypes appear to be the only conserved family identified, with classical branching factor homologues Arp 2 and 3 not identifiable through homology searches in many apicomplexan parasites [166]. *Plasmodium* expresses two classical formins (UniProt AC: A0A509AMX5, A0A509AUT2) and one formin‐like protein called MISFIT (UniProt AC: A0A509ANE6), while *Toxoplasma* expresses three classical formins (UniProt AC: D0V3Y0, D0V3Y1, B6KTA3). The classical formins contain classical FH1 and FH2 domains, although interestingly apicomplexan formin 1 has a much reduced FH1 domain in comparison to both classical formins and formin 2. They also curiously lack typical regulatory features such as the GTPase‐binding and diaphanous auto‐regulatory domains [167]. Yet, *Plasmodium* formins display expanded N‐terminal tetratricopeptide repeat domains (for formin 1) or a single N‐terminal PTEN‐C2‐like domain [108]. Both parasite formins are nucleators of actin and filament elongators as their classical homologues, but the roles of each formin are not the same in apicomplexans. Formin 1 is a more efficient nucleator *in vitro* and plays a major role in contributing to host cell invasion and motility [167]. This is reflected in its localisation, where it is located close to other molecules of the invasion machinery [137, 167]. Formin 2, on the other hand, is important for parasite cell division, the elongation of sexual cells and the inheritance of a parasite‐specific organelle called the apicoplast [108, 168, 169, 170]. This role has been shown for both *Toxoplasma* and *Plasmodium*, a striking observation given that the mechanisms of cell division are fundamentally different: *Toxoplasma* undergoes endodyogeny (the formation of two daughter cells within a single mother cell) while *Plasmodium* blood stages under schizogony (the formation of multiple nuclei in a common cytoplasm and with a final synchronous cytokinetic event to produce multiple daughter cells). This highlights that such divergent mechanisms still have actin at their heart and also a set of common regulators. Each formin has thus developed specialist functions for the specialist requirements of apicomplexans.

**Fig. 4:**
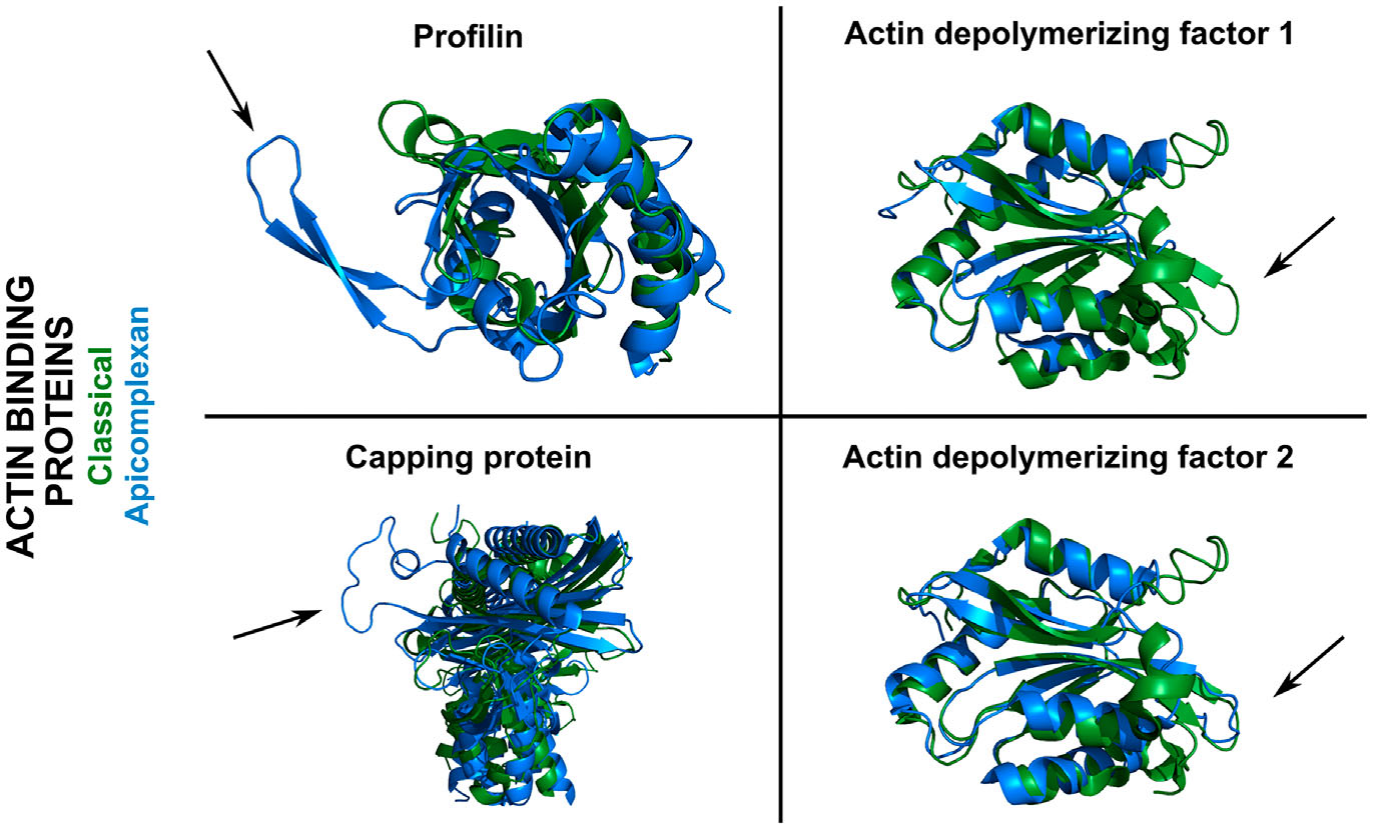
Apicomplexan actin‐binding proteins display divergent structural features in comparison to classical homologues. Respective apicomplexan actin‐binding proteins (blue) were structurally aligned to classical counterparts (green). Selected divergent features for each complex are indicated with a black arrow. Apicomplexan profilin possesses an extended beta‐hairpin arm. Actin‐depolymerising factor 1 lacks the classical F‐actin‐binding loop while actin‐depolymerising factor 2 has a typical loop in this position. The alpha homodimer of apicomplexan capping protein has a loss of canonical interaction sites and, shown here, a unique insertion that possibly mediates unique contacts with parasite actin filaments. Capping protein dimer shown as side view. Please note that, for comparison purposes, the C‐terminal helix of classical capping protein is not shown as the equivalent apicomplexan C terminus was experimentally truncated for crystallisation studies. Structures rendered in open‐source PyMOL. PDB IDs used are as follows: Profilin: 1fil (*H. sapiens*) and 2jkf (*P. falciparum*); actin‐depolymerising factor 1: 4bex (*H. sapiens*) and 2xf1 (*P. berghei*); actin‐depolymerising factor 2: 4bex (*H. sapiens*) and 2xfa (*P. berghei*); Capping protein (alpha): 8f8q (*H. sapiens*, αβ dimer) and 7a0h (*P. berghei*, αα homodimer).

Most ABPs regulating monomer treadmilling such as profilin, CAPs and actin‐depolymerising factors are present in model apicomplexans, yet clear homologues of thymosin‐beta and gelsolins have not been identified [166]. This is perhaps the least surprising, given that the majority of cellular apicomplexan actin is considered to be in the monomeric state [171]. Profilin (UniProt AC: Q58NA1, P86294) is essential for parasite gliding and host cell invasion and can stimulate immune responses, for example, via toll‐like receptor TLR11 [172]. Interestingly, in contrast to classical systems, apicomplexan profilins function in monomer sequestration rather than feeding actin monomers to formins and the growing filament, and also inhibit nucleotide exchange [99, 173, 174] (Table 1), pointing to a role in keeping actin polymerisation in parasites to a necessary minimum. Apicomplexan profilins have a very similar core in comparison to classical profilins, but have a conserved additional insertion that forms an arm‐like β‐hairpin structure [175] (Fig. 4) and forms an additional interface with the actin monomer. Mutation of this arm showed that this region has crucial interactions through an additional unique actin‐binding site that is important for efficient parasite motility [173]. *Plasmodium* profilin also has a unique acidic loop that is important for force production and retrograde flow in the parasite [176]. This indicates that profilin has developed specialised interactions to regulate specific actin dynamics in these parasites. However, this also shows the types of interactions needed that can differentiate between actin binding and delivery to a growing filament, a basic feature of actin biology.

Apicomplexan CAPs are much smaller than their multi‐domain classical counterparts and contain only the C‐terminal X‐linked retinitis pigmentosa two protein (CARP) β‐sheet domain. Apicomplexan CAPs, even in this truncated form, can dimerise, bind actin monomers directly and mediate nucleotide exchange, revealing the minimum domain structure required for this highly conserved mechanism [177, 178] (Table 1). Gene deletion or overexpression in *Plasmodium* revealed roles of CAP (UniProt AC: Q8I288) in mosquito infection of the midgut or the salivary glands, respectively [177, 179]. *Toxoplasma* has two isoforms expressed from the same gene (UniProt AC: A0A125YN74) that localise in different cellular locations. Depletion of CAP in *Toxoplasma* affects multiple processes including cell invasion, egress, motility, correct daughter cell orientation and trafficking [180]. Interestingly, apicoplast inheritance, a process that formins are important for, was not affected by deletion of *Toxoplasma* CAP, highlighting the differential spatial requirements of actin and its regulators in various cellular functions.

Apicomplexan ADFs are highly divergent, whereby they are <40% identical in sequence identical in comparison to classical forms. *Plasmodium* expresses two *ADF* genes (UniProt AC: Q8I467, Q3YPH0), while the *Toxoplasma* genome, like yeast, only contains one (UniProt AC: O15902). The two ADFs in *Plasmodium* are even more sequence divergent from each other (<30% sequence identity) than they are from classical ADFs [181]. *Plasmodium* ADF1 is one of the shortest known ADFs, measuring 122 amino acid residues [182]. Knockout studies in *Plasmodium* indicate that the two different ADFs appear to have different degrees of importance, with ADF1 being essential in blood stages while the deletion of ADF2 had no influence at this stage [182] and only a minor effect in transmission to mosquitoes for the stages already analysed [183]. The single ADF in *Toxoplasma* resembles that of ADF1 in *Plasmodium* in sequence and structure. Inducible knockout leads to strong decreases in invasion and motility of *Toxoplasma* parasites and also exit of the parasites from host cells [184]. *Plasmodium* ADF1 and *Toxoplasma* ADF possess a shortened F‐loop in comparison to classical ADFs [179] (Fig. 4), which has consequences for its function. *Plasmodium* ADF1 only binds actin monomers, stimulates nucleotide exchange and does not readily bind to actin filaments [182, 184, 185] (Table 1). *Plasmodium* ADF2 has a more developed F‐loop (Fig. 4) and thus more readily binds and severs filaments in comparison to ADF1 yet, similar to ADF1, also stimulates nucleotide exchange rather than inhibiting this process [181].

Filament capping proteins have been identified in apicomplexans, yet *Plasmodium* and *Toxoplasma* only express one isoform of each dimer subunit (UniProt AC: A0A509AR49, A0A509AQN8, A0A125YI27, S8EN02). Apicomplexan capping proteins are capable of capping actin filaments [186] and control polymerisation atypically by reducing filament length without affecting the critical concentration [187] (Table 1). Even more striking, while other organisms typically only have functional capping proteins in the heterodimeric form (i.e. a quaternary structure containing one alpha and one beta subunit), apicomplexan capping protein alpha subunit seems to have evolved unique and to some extent independent functions [186, 188]. The alpha and beta subunits display different expression profiles and different phenotypes when their respective genes are deleted. The beta subunit has lower expression in blood stage parasites and deletion of the gene does not affect blood parasite growth yet is important for the initial infection of mosquitoes, likely in its more classical heterodimeric state. The alpha subunit is required for blood stages and is capable of binding to actin in the absence of the beta subunit, indicating a functional competency as a single subunit. This function is fulfilled in a homodimeric state, which results in a noncanonical, asymmetric dimeric structure, highly uncommon for proteins in general. The altered structure of the alpha subunit appears to result in a loss of canonical interaction sites but also contains unique insertions that could facilitate binding to parasite actin [187] (Fig. 4). This highlights the breadth of function of these capping proteins and the unique needs of the parasite actin cytoskeleton. Indeed, the presence of a *Toxoplasma*‐unique actin‐binding protein, toxofilin, which binds to actin dimers and caps filaments [21, 189] indicates specialist needs of the parasite.

Coronin (UniProt AC: Q5Y1E7, A0A509ARM7) appears to be the only apicomplexan ABP classified as an actin filament crosslinker or bundler, with cytoskeletal structural proteins (e.g. talins and vinculin) also not identified through homology searches. Apicomplexan coronins resemble type 1 mammalian coronins, which are typically smaller than other family members. This type contains a single highly conserved WD40/beta propeller domain and downstream conserved regions at the N terminus, in addition to unique and coiled coil domains of variable lengths at the C terminus. Dimerisation of coronin has been shown to be mediated, as classically observed, through the coiled coil domains in *Toxoplasma* coronin, with this feature likely conserved in other apicomplexans [190]. Biochemically, apicomplexan coronin can bind actin filaments but has relatively weak bundling activity in comparison to classical homologues [190, 191, 192] (Table 1). Coronin is localised towards the back of moving parasites and, while dispensable for parasite replication, is important for directional motility and invasion of these parasites [190, 193]. Interestingly, mutations of coronin have also been identified as conferring reduced susceptibility to the frontline antimalarial treatment, artemisinin, likely due to a function in haemoglobin uptake by the human‐infecting malaria parasite [194, 195].

It is also worth emphasising that while apicomplexans likely have fewer ABPs than higher eukaryotes, it is also entirely possible that unique ABPs have developed to fulfil the demands of regulating the highly divergent actin in these systems, as has already been observed for Toxofilin. Indeed, the nonapicomplexan protist *Giardia* did not have predicted classical ABPs based on genome searches [107], but further protein–protein interaction investigations have indicated the presence of ABPs [196, 197]. Such findings could be generally expected from other minimalist parasites such as apicomplexans and identify novel regulators of eukaryotic actin. Indeed, a recent phenotypic screen in *Toxoplasma* was able to identify at least two proteins that might be indirectly involved in actin dynamics or localisation at particular steps in parasite exit from its host cell [198]. Further, pulldown with the only conserved member of the Arp2/3 complex subunit in *Plasmodium* (ARPC1) identified a complex that functions in a noncanonical manner to mediate DNA segregation [199].

## Conclusion

Eukaryotes are a broad group of organisms of which only a small minority are actively researched, with even less being done so with the latest genetic toolkits. Apicomplexan parasites represent a good example of the knowledge gains of studying nonclassical, highly divergent eukaryotes and their actin cytoskeletons. Understanding the divergent nature of actin and its binding proteins provides deeper insight of the spectrum of both the core function and the biochemical breadth of eukaryotic actin biology. Further in‐depth studies of both apicomplexans and other eukaryotes, both more extensive and underexplored, will continue to shed light on the remarkable biochemical properties of actin and its fundamental role in cell biology.

## Conflict of interest

The authors declare no conflict of interest.

## Author contributions

Both authors wrote the manuscript and prepared the figures and table.
